# Dr. William H. Draper

**Published:** 1901-05

**Authors:** 


Dr. William H. Draper, of New York, one of the most prominent physicians in this country, died at his residence in that city, Friday, April 26, 1901, after a somewhat prolonged period of ill health. Dr. Draper has been prominent as a writer and teacher for thirty years or more, and was justly regarded as a leader in medical thought.

